# Dengue periodic outbreaks and epidemiological trends in Nepal

**DOI:** 10.1186/s12941-018-0258-9

**Published:** 2018-02-23

**Authors:** Birendra Prasad Gupta, Reshma Tuladhar, Roshan Kurmi, Krishna Das Manandhar

**Affiliations:** 1 Virology Unit, Central Department of Biotechnology, Tribhuvan University, Kirtipur, Kathmandu, Nepal; 2 Central Department of Microbiology, Tribhuvan University, Kirtipur, Kathmandu, Nepal; 3 Bhawani Hospital, Birgunj, Parsa, Nepal; 4 Central Diagnostic Laboratory and Research Center Pvt. Ltd, Kathmandu, Nepal

**Keywords:** Dengue, Nepal, Epidemic, Outbreak

## Abstract

Dengue is a global health problem and expansion of its endemics towards new territories in the hilly regions in Nepal is a serious concern. It appeared as a new disease in Nepal in 2004 from Japanese traveler with sporadic cases every year and massive outbreaks in 2010, 2013 and 2016. The serotype was responsible for outbreak in particular year was dengue virus serotype-1 (DENV-1) in 2010, 2016; and DENV-2 in 2013. Nepal lacks basic health related infrastructure in rural areas and does not have a stringent health care policy. With severances of epidemic like dengue, a new surveillance or an upgrading of existing one are direly needed to better challenge the possible outbreaks. This review paper aims to explain the dengue trend in last one decade in Nepal and warrants concerted and timely public health interventions to minimize the deleterious effects of the disease.

## Background

Dengue is considered as one of the predominant arboviral infection and is caused by one of the 4 serotypes of the dengue virus (DENV 1-4). It is a RNA virus that belongs to the genus Flavivirus of the family *Flaviviridae* [1]. Generally, dengue fever (DF) is a self-limiting disease without any long-term effects after the fever subsides. However, dengue hemorrhagic fever (DHF)/dengue shock syndrome (DSS) is a life-threatening disease; characterized by increased vascular permeability; which may lead to hypovolemic shock, hemoconcentration, haemorrhages, thrombocytopenia, pleural effusion, and possibly even death [2]. In South Asia, over 76% of infected people are asymptomatic and 24% of the infected population show distinct clinical symptoms [3]. The first case of dengue in Nepal was reported from a Japanese traveler after returning to his country in 2004 [4]. Although several cases of dengue fever were previously suspected in Nepal, a scientific documentation as case report of dengue from the indigenous Nepali population was only published after 2006 [4, 5]. Since then, sporadic clinical cases of dengue and outbreak has been reported every year in the country [6–8]. Among the two competent vectors of the disease *Aedes aegypti* and *Aedes albopictus*, the former is the primary vector for transmission among humans and is distributed only in the lowland Terai region of Nepal, whereas *A. albopictus* is found throughout Nepal [8]. Although *A. albopictus* was reported in the southern plains during the 1980s, *A. aegypti* was first reported in Kathmandu in 2009 [9]. The aim of this review paper is to summarize the epidemiological pattern/serotype shift of dengue virus infections in Nepal since last decade and emphasize to improve the diagnostic facilities/capacities to encounter maximum number of cases and public health interventions programme to minimize the deleterious effects of the disease in Nepal.

## Methods

The data are acquired by reviewing the previously published paper and annual report of Department of Health Services (DOHs), Ministry of Health, Government of Nepal. We found the dengue cases were screening by using commercially available rapid diagnostic kit (RDT) and conformation was done using ELISA (either dengue NS1 and/or IgM positive) at the respective hospitals. The data was entered and analyzed (mean, median and percentage) using SPSS software version 23.0.

### Dengue epidemics in Nepal

**Fig. 1:**
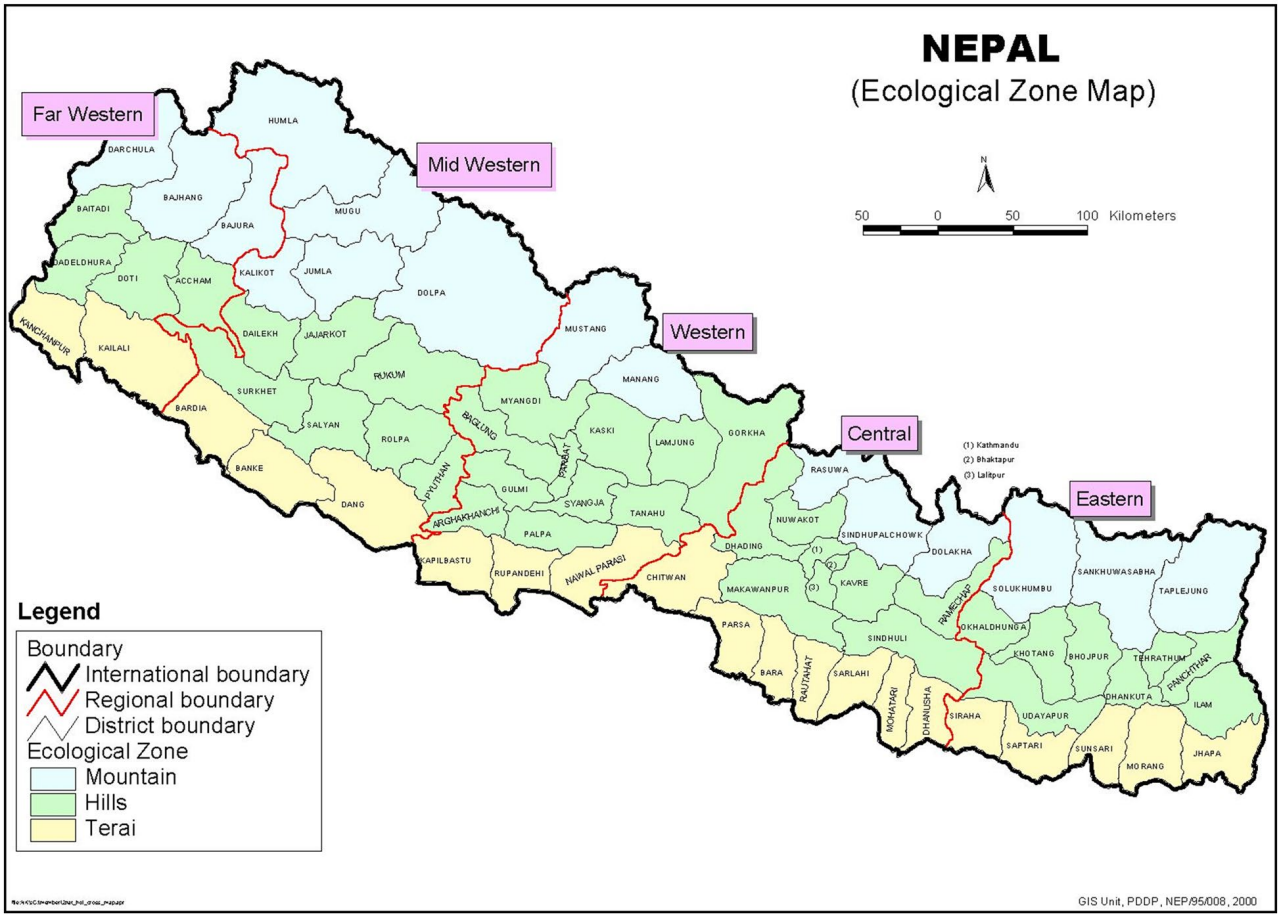
Map of Nepal with Ecological Zone

**Fig. 2:**
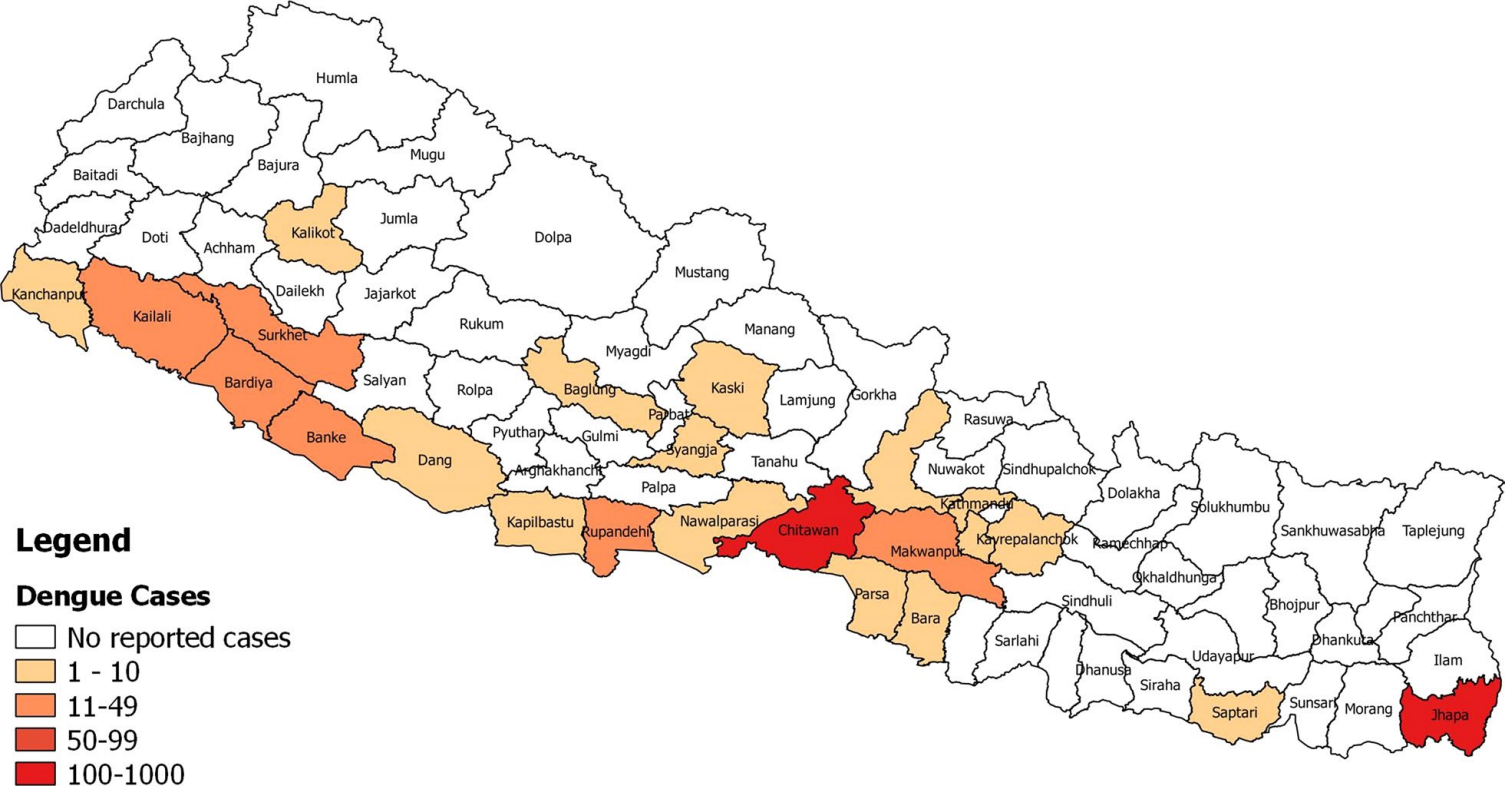
Map of dengue reported districts from 2006 to 2016 in Nepal

Nepal is a landlocked country situated in the central Himalayas area of South Asia. It is known for its three particular ecological zones: the northern range-Mountain; the mid-range-Hill region; and the southern range-Terai (“flat land”) (Fig. 1). A little more than half of Nepal’s present population dwells either in a tropical or in a subtropical atmosphere of Terai, where all dengue episodes have occurred and the first documentation of indigenous dengue in Nepal was reported from the Terai region of Nepal [10]. Since then, sporadic cases and/or outbreaks continued validating DENV epidemics in the country [11]. The dengue-wave spread from Terai region and was detected every year in that particular Terai region. The Chitwan and Rupandehi districts in the Terai region of Nepal were focal epidemics during the outbreak in 2010, 2013 and 2016 [10, 12, 13]. Not only in the lowland inner Terai region (Parsa district), which is 300 m below sea level, DENV have been reported in the valleys of upland Hill regions at an altitude of 2500 m above sea level [14] (Fig. 2).

### Clinical versus confirmed dengue cases

Suspected clinical cases presenting to the hospitals were 3–8 times higher in number than the confirmed dengue patients. Up to 2016, the reported total numbers of confirmed cases in the country were 3634 among 10,966 clinical cases (Table 1). The highest number of clinical cases (n = 4125) occurred during the outbreak in 2016 and the lowest (n = 25) in 2008. However, percentage of confirmed cases was found to be highest (75.47%) in 2010 which abruptly decreased to 14.1% in 2011 and increased again to 39.15% in 2016 (Table 1).

**Table 1:** Epidemiological profile of dengue in Nepal from 2006 to 2016

Parameters	2006^a^	2007^b^	2008^b^	2009^b^	2010^a^	2011^b^	2012^b^	2013^a^	2014^b^	2015^b^	2016^a^
Number of dengue cases											
Clinical dengue	276	100	25	35	1215	562	1167	2340	576	545	4125
Confirmed dengue	32 (11.6%)	27 (27%)	10 (40%)	30 (85.71%)	917 (75.47%)	79 (14.1%)	183 (15.7%)	642 (27.43%)	73 (12.8%)	26 (4.7%)	1615 (39.15%)
Serotype detected											
Serotype	1, 2, 3, 4	ND	ND	ND	1	ND	ND	2	2	ND	1
Gender of confirmed dengue											
Male	24	17	6	21	530	51	114	456	52	17	960
Female	8	10	4	9	387	28	69	186	21	9	355
Affected region											
Terai	5	1	2	3	8	15	11	9	5	6	15
Hill	0	0	1	2	4	0	0	5	2	2	3
Age wise distribution of conformed dengue cases (years)											
< 15	6 (18.8%)	14 (51.8%)	5 (50%)	13 (43.3%)	345 (37.6%)	3 (3.8%)	11 (6.0%)	234 (36.4%)	11 (15.1%)	6 (7.8%)	145 (11%)
15–40	22 (68.8%)	6 (22.2%)	3 (30%)	11 (36.7%)	234 (25.5%)	71 (89.9%)	163 (89.1%)	378 (58.8%)	57 (78.1%)	15 (57.6%)	876 (66.6%)
> 40	4 (12.5%)	7 (25.9%)	2 (20%)	6 (20%)	338 (36.8%)	5 (6.3%)	9 (4.9%)	30 (4%)	5 (6.8%)	5 (19.2%)	294 (22.3%)

^a^Outbreak

^b^Sporadic

### Gender and age based susceptibility

The number of the infected male (n = 2248) population was significantly higher than the female (n = 1086) at the ratio of 1.17:2.5 (Table 1). The age group of 15–40 years (median age 27.35) was distinctly vulnerable, however, affected patients ranged from a 2 year old child to 87 year old man. The percentage of children suspected, below 15 years, was found high in 2007 and 2009 as 51.8 and 43.3% respectively. The proportion of confirmed infections in children less than 15 years of age was two earlier outbreaks (2010, 2013), which continued in the year 2016 as well. Population more than 40 years of age was comparatively less infected to other studied group.

### Circulation of serotype

Serotyping of the virus was not conducted every year, though the first reported case from the Japanese traveler in 2004 was recorded as serotype-2 [15]. The circulation of multiple serotypes in the country was identified during the 2006 outbreak [16]. However, different serotypes predominated every year with DENV-1 in 2010 and 2016, DENV-2 in 2013 (Table 1).

### Drift of dengue virus from subtropical towards temperate zones

The first outbreak of dengue fever was documented from Terai region of central part in 2006 [17]. Unexpectedly, dengue erupted in 2010, 2013 and 2016 covering the whole west to east Terai region encompassing middle Hill districts (Annual Report Department of Health, EDCD, 2016). The climate in Nepal though, situated geographically in sub-tropical climate zone, ranges from tropical to alpine. The dengue cases were no more limited to subtropical areas but have crossed the climatic border and extended towards hill regions enduring the temperate climate.

### Dynamics of dengue vector in Nepal

Numerous factors are responsible for the dengue transmission in the country and one of it is believed to be associated with the spread of the vector within the country. Though rapid urbanization and increasement of travel have been obvious in the country, the climate conditions have also been projected as a contributional factor to the spread of the dengue vector. A stable population of both *A. aegypti* and *A. albopictus* have been found to be established from the lowlands to the middle mountain at an altitude of 2000 m above sea level in Nepal [18–20]. This scenario makes it likely that Hill regions in Nepal will continue to be environmentally favored for the breeding of dengue vector and consecutively may constitute a potential risk for dengue outbreaks in the future.

## Discussion

Our analysis of the dengue epidemiology in Nepal showed a substantial increase of the dengue virus prevalence over a short span of time. Although the first dengue case was reported in 2004 from Japanese traveler, the outbreak of dengue was occurred in the country in 2006 with a remarkable number of 32 confirmed cases [16, 21]. The virus remained almost latent for the three consecutive years from 2007 to 2009 and reoccurred again during a massive outbreak in 2010 [10]. Following this outbreak, cases of dengue continued to be reported in the subsequent year 2011 and 2012 and two major outbreaks were witnessed in 2013 and 2016 (Annual Report Department of Health, EDCD, 2016). A clear cyclic 3-year-amplitude demonstrated by major peaks in 2010, 2013 and 2016. Such epidemiological cyclic outbreak-trend every 3 years have also been experienced in Brazil and Cuba [22, 23].

Annual seasonal variation trends of the dengue occurrence shows, infections appear abruptly in July, just after the start of the rainy season and cases peaks in August and September, which are considered to be the months with the most favorable climate for mosquitoes breading [24, 25]. Similar seasonal response was found in a study conducted in China and Philippines [26, 27]. Similarly, peak transmission in the post monsoon season supports similar findings from other studies conducted in India [28]. The distinct seasonal trend of dengue prevalence/infections in Nepal needs to be considered when addressing precautions and public awareness programs during the specific months in order to control possible future outbreaks.

The study for 11 years with regards to the gender based analysis showed that the male population was more likely to have infection than female at the mean ratio of 2:1, which is in agreement to the past study done by Gupta et al. [25]. One can assume that compared to women who are generally confined within household works the male population is mostly involved in outdoor work activities for livelihood which makes them more likely exposed to *Aedes* spp. bites.

Nepal shows heterogeneity of multiple dengue serotypes as circulation of all four serotypes was found in 2006. Since then, specific serotypes were predominant in 2010 and 2016 outbreak (DENV-1) and 2013 (DENV-2). Similar findings on serotype prevalence was seen in Delhi, India showing circulation of all four serotypes in 2003 followed by the predominance of serotype 3 in 2004 and 2005 [28]. The sub-metropolitan cities of lowland Terai region of Nepal are densely populated and share open transit multiple routes to North India facilitating easy transmission and import of cases, especially following outbreaks in India. This region of Nepal is considered highly vulnerable to dengue outbreaks. However, since the subtropical climate favors the dengue vector, the middle mountain region has also potential for dengue outbreaks.

## Conclusion

This review paper summarizes the dengue epidemiology in Nepal chronologically including seasonal influence, age and gender distribution. The dengue virus serotype shift observed in each outbreak appears as a major factor in the dengue landscape in Nepal. These shifts may enhance the disease severity and complications in the future and requires concerted and timely public health interventions to protect the population at risk in Nepal.

## Abbreviations

DF: dengue fever
DHF: dengue hemorrhagic fever
DSS: dengue shock syndrome
DENV-1: dengue virus serotype-1
DENV-2: dengue virus serotype-2
DENV-3: dengue virus serotype-3
DOHs: Department of Health Services
ND: not done
